# Clade-Specific Plastid Inheritance Patterns Including Frequent Biparental Inheritance in *Passiflora* Interspecific Crosses

**DOI:** 10.3390/ijms22052278

**Published:** 2021-02-25

**Authors:** Bikash Shrestha, Lawrence E. Gilbert, Tracey A. Ruhlman, Robert K. Jansen

**Affiliations:** 1 Department of Integrative Biology, University of Texas at Austin, Austin, TX 78712, USA; lgilbert@austin.utexas.edu (L.E.G.); truhlman@austin.utexas.edu (T.A.R.); jansen@austin.utexas.edu (R.K.J.); 2 Center of Excellence in Bionanoscience Research, Department of Biological Sciences, Faculty of Science, King Abdulaziz University, Jeddah 21589, Saudi Arabia

**Keywords:** heteroplasmy, interspecific cross, *Passiflora*, plastid, plastid segregation/sorting-out, restriction digestion, uniparental and biparental inheritance

## Abstract

Plastid inheritance in angiosperms is presumed to be largely maternal, with the potential to inherit plastids biparentally estimated for about 20% of species. In *Passiflora*, maternal, paternal and biparental inheritance has been reported; however, these studies were limited in the number of crosses and progeny examined. To improve the understanding of plastid transmission in *Passiflora,* the progeny of 45 interspecific crosses were analyzed in the three subgenera: *Passiflora, Decaloba* and *Astrophea.* Plastid types were assessed following restriction digestion of PCR amplified plastid DNA in hybrid embryos, cotyledons and leaves at different developmental stages. Clade-specific patterns of inheritance were detected such that hybrid progeny from subgenera *Passiflora* and *Astrophea* predominantly inherited paternal plastids with occasional incidences of maternal inheritance, whereas subgenus *Decaloba* showed predominantly maternal and biparental inheritance. Biparental plastid inheritance was also detected in some hybrids from subgenus *Passiflora.* Heteroplasmy due to biparental inheritance was restricted to hybrid cotyledons and first leaves with a single parental plastid type detectable in mature plants. This indicates that in *Passiflora*, plastid retention at later stages of plant development may not reflect the plastid inheritance patterns in embryos. *Passiflora* exhibits diverse patterns of plastid inheritance, providing an excellent system to investigate underlying mechanisms in angiosperms.

## 1. Introduction

Plant cells house three genome-containing organelles: the nucleus, the mitochondrion and the plastid. Unlike the nuclear genome, both the mitochondrial and plastid genomes (plastomes) are inherited in a non-Mendelian fashion. Plastid inheritance is usually reported as uniparental, and mixed genotypes do not persist as plastids undergo vegetative segregation. The stochastic process of partitioning during plastid and cell division usually results in plastome homogeneity (homoplasmy) early in plant development [1,2,3,4]. Taken together, these genetic features suggest plastomes are generally homogenous despite the presence of many unit genomes per nucleoid, nucleoids per plastid and plastids per cell [5].

It is generally accepted that plastid inheritance is maternal among angiosperms, whereas paternal inheritance is common among gymnosperms, with exceptions in both groups [1,6,7,8,9]. Cytological screenings of pollen for plastid DNA (ptDNA) of nearly 300 angiosperm species reported that approximately 80% and 20% have the potential for maternal and biparental plastid transmission, respectively [7,8]. The bias toward maternal inheritance is attributed to the distribution of plastids during pollen development. During mitotic divisions of the microspore, the paternal plastid is excluded from the sperm cell [10,11]. Other mechanisms that have been described prevent paternal plastid transmission during and post fertilization, consequently reinforcing maternal plastid transmission [11,12,13].

The prevalence of maternal inheritance in angiosperms may be driven by constraints to intracellular conflict and the spread of selfish elements associated with paternal plastids [14,15,16]. However, uniparental inheritance systems can lead to the accumulation of deleterious mutations over evolutionary time. To overcome this, biparental inheritance may have evolved as a mechanism to rescue defective plastids and alleviate mutational load [15,16,17]. In the past few decades, plastid inheritance studies have expanded in many plant lineages, and evidence of paternal leakage and biparental inheritance has been reported in lineages that were thought to exhibit strictly maternal inheritance [18,19,20]. This could be taken as a support for the hypothesis that paternal transmission helps to prevent the perils of strictly maternal inheritance, but empirical evidence to support this is lacking. Nonetheless, these studies indicate that the underlying mechanisms that restrict paternal plastid transmission are not completely effective.

Initially described by Baur (1909) based on variegated phenotypes in *Pelargonium* [21], biparental plastid inheritance has been identified in a few other lineages, including *Campanulastrum* [17], *Oenothera* [22], *Medicago* [23,24,25], *Turnera* [26,27], *Zantedeschia* [28] and *Passiflora* [29]. Two lineages, *Pelargonium* and *Oenothera*, have been extensively studied, and variations in plastid transmission patterns were shown to be under the strong influence of nuclear or plastid genomes [1,22,30]. With an increasing number of progeny being assayed in inheritance studies, low frequency paternal transmission has been detected in lineages that were previously considered to utilize strict maternal inheritance [18,31,32,33]. Low frequency paternal inheritance is common in interspecific crosses and may result from a failure of mechanisms that evolved to prevent it [14,29,34,35]. Two lineages of rosids, *Medicago* and *Turnera*, exhibit rare predominant paternal inheritance even in intraspecific crosses, a pattern that is commonly observed among gymnosperms [24,26,27,36]. Predominant paternal plastid inheritance with occasional biparental and maternal inheritance noted in these two lineages is in contrast with general patterns documented among angiosperms.

The use of ptDNA has been critical in phylogenetic inference and has numerous applications in plant research, including biotechnology, breeding, conservation and biogeography [37,38]. However, exceptions in inheritance patterns that include biparental inheritance and occasional paternal leakage could be problematic for phylogenetic inference. When plastome variation exists between parents, paternal leakage or biparental transmission could result in heteroplasmy, a condition in which different plastome types are present in a cell or in an individual. In such cases, if the plastome variation is substantial, heteroplasmy could introduce complications of paralogy, thereby perturbing the inference of phylogenetic history [39]. The implications of heteroplasmy in phylogeny reconstruction have been documented in several lineages, including *Hyobanche* [39,40] and *Passiflora* [41]. In these cases, conflicting phylogenetic relationships were inferred based on the sampling of heterologous loci. It should be noted that several other processes, such as gene duplication and transfer, could also contribute to apparent heteroplasmy [39,42]. Nonetheless, recognizing patterns of plastid inheritance could help to achieve correct conclusions when using ptDNA to estimate phylogenetic relationships [39,43].

Plastid inheritance studies in *Passiflora* have reported uniparental (paternal or maternal) and biparental inheritance. *Passiflora* (*P.*) is the largest genus in Passifloraceae and includes about 560 species grouped into five subgenera, *Astrophea, Decaloba, Deidamioides, Passiflora* and *Tetrapathea,* with subgenera *Passiflora* and *Decaloba* each containing more than 200 species [44,45,46,47]. The cytological study by Corriveau and Coleman (1988) included a commonly cultivated species *P. edulis* (subgenus *Passiflora*) that exhibited the potential for biparental plastid transmission [7]. Based on three interspecific artificial crosses and one natural hybrid, Muschner et al. (2006) found that hybrids within subgenus *Passiflora* inherited paternal plastids, whereas those in subgenus *Decaloba* inherited maternal plastids, suggesting distinct inheritance patterns between the subgenera [48]. Hansen et al. (2007) expanded the number of interspecific hybrids from subgenus *Passiflora* and also included crosses between conspecifics from geographically isolated populations [29]. The expanded interspecific crosses and progeny supported paternal plastid inheritance in subgenus *Passiflora* (17 paternal), but occasional biparental inheritance with predominately maternal inheritance (12 maternal, 3 biparental) was detected in intraspecific hybrids. The authors suggested a dichotomy of paternal and maternal inheritance in *Passiflora* between inter- and intraspecific crosses, respectively. The correlation of maternal inheritance with intraspecific crosses was based on only two hybrid crosses from subgenera *Passiflora* and *Decaloba, P. pseudo-oerstedii* × *P. oerstedii* and *P. costaricensis* from two locations, respectively [29]. *Passiflora pseudo-oerstedii* is considered a distinct species *P. dispar* [49]; therefore, in Hansen et al., *P. pseudo-oerstediiP. oerstedii* was not an intraspecific hybrid cross. Thus, only a single intraspecific cross from subgenus *Decaloba* was performed and showed maternal plastid inheritance. The conclusion that maternal inheritance is correlated with intraspecific crosses in *Passiflora* is tenuous since it is based on a single observation.

Biparental inheritance in *Passiflora* has also been reported in interspecific hybrids from subgenus *Passiflora* in crosses involving *P. menispermifolia* and *P. oerstedii,* which displayed a hybrid-bleaching phenotype resulting in hybrid lethality likely due to plastome-genome incompatibility [50]. The remarkable difference in inheritance patterns observed in *Passiflora* suggests that multiple mechanisms may be involved in plastid inheritance. However, due to the limited number of crosses and progeny examined, the extent of variation in inheritance patterns remains uncertain. Considering the species diversity in the genus, comprehensive sampling would improve the understanding of the diversity of plastid inheritance patterns in *Passiflora.* In the present study, plastid inheritance was examined in 45 interspecific crosses in subgenera *Passiflora, Decaloba* and *Astrophea.* Polymerase chain reaction (PCR) amplification of ptDNA followed by restriction endonuclease (RE) digestion of amplicons was used to detect the plastid types in the hybrids. Plastid inheritance in *Passiflora* hybrids was examined in embryos and at different stages of plant development to better understand inheritance and retention of plastid types at different phases of the plant life cycle.

## 2. Results

**Figure 1:**
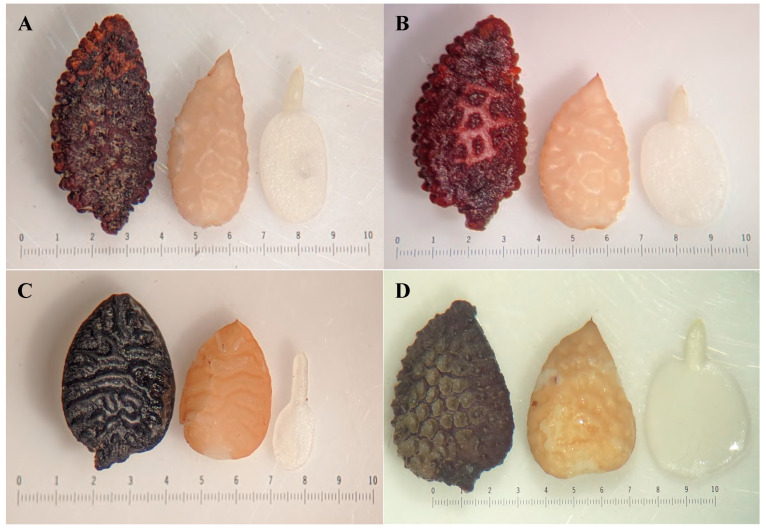
*Passiflora* hybrid seeds under dissecting microscope at 10× magnification. (**A**) *P. menispermifolia* (8039) × *P. menispermifolia* (Sirena) progeny for comparison with interspecific hybrid in B that includes same maternal parent. (**B**) *P. menispermifolia* (8039) × *P. miersii* from subgenus *Passiflora*. (**C**). *P. rufa* × *P. auriculata* from subgenus *Decaloba*. (**D**) *P. sphaerocarpa* × *P. pittieri* from subgenus *Astrophea*. Left to right, seeds soaked in water for 24 h, seed without seed coat and embryo only.

Crosses were considered successful only when the pollen transfer onto the stigma produced a fruit that contained seeds with embryos (Figure 1). With this criterion, the number of successful crosses was substantially lower than the number of attempted crosses (>100). *Passiflora* hybrids were named following the standard nomenclature for hybrids (maternal species × paternal species). Forty-five crosses from the three subgenera, *Passiflora, Decaloba* and *Astrophea*, were examined. Most successful crosses (37) occurred within subgenus *Passiflora* and included four reciprocal crosses (Table 1). Seven successful crosses were examined in subgenus *Decaloba,* two of which were reciprocal crosses. Subgenus *Decaloba* includes hybrid crosses between two accessions of *P. misera*. *Passiflora misera* (9023) has notably flattened stems with flowers similar to those commonly described as *P. misera*, whereas *P. misera* (9335) has square stems and morphologically distinct flowers. Therefore, the two accessions of *P. misera* were considered separate species and, accordingly, their hybrids were considered interspecific. Plant vouchers for *P. misera* and their hybrids were deposited in the University of Texas herbarium (TEX-LL, Supplementary Table S1, see Supplementary Materials with this article). In subgenus *Astrophea,* a single interspecific cross was successful. Multiple attempts to generate hybrids between species in subgenus *Deidamioides* were unsuccessful.

Several ptDNA target regions were selected for PCR and RE analysis, with the *rpl32-trnL* region being the most useful for assessing plastid types. Detailed information on *Passiflora* species used to generate hybrids, the direction of crosses, ptDNA target regions, accession numbers for sequences, primers used to amplify target regions, REs and vouchers are provided in Supplementary Tables S1–S4.

### 2.1. Plastid Inheritance in Hybrid Embryos

Substantial differences in embryo size were noted between hybrids in different subgenera. The embryos in subgenus *Decaloba* hybrids were generally much smaller compared to hybrid embryos in subgenera *Passiflora* and *Astrophea* (Figure 1). Direct PCR using crude extract from hybrid embryos successfully amplified ptDNA target regions for most of the crosses except three, *P. nephrodes* × *P. oerstedii*, *P. lancetillensis* × *P. microstipula* and *P. microstipula* × *P. lancetillensis*. For the progeny of these three crosses, DNA was isolated from embryos prior to PCR amplification. No amplification products from embryos of the *P. nephrodes* × *P. oerstedii* were generated even after DNA isolation; therefore, the cross was excluded from further analyses.

Plastid inheritance in the *Passiflora* hybrid embryos may be classified as purely paternal, purely maternal, biparental or a combination of any of the three patterns (Table 1). In subgenus *Passiflora,* hybrid embryos exhibited uniparental (paternal or maternal) and biparental patterns of plastid inheritance (Table 1, Supplementary Figures S1 and S2). For the 37 interspecific crosses examined, hybrid progeny from 24 crosses displayed solely paternal inheritance, while progeny from six crosses showed maternal and/or biparental in addition to paternal inheritance (Table 1). The remaining seven crosses in subgenus *Passiflora* displayed maternal and/or biparental inheritance. Solely maternal transmission was found in two hybrid crosses, *P. menispermifolia* (9224) × *P. oerstedii* and *P. nephrodes* × *P. choconiana*; however, their reciprocal crosses had exclusively paternal inheritance (Table 1): plastids from *P. menispermifolia* and *P. nephrodes* were inherited regardless of the direction of the crosses. Solely biparental plastid inheritance was detected in two crosses, *P. oerstedii* × *P. menispermifolia* (8039) and *P. hastifolia* × *P. menispermifolia* (8039). Of the total 357 embryos examined from 37 interspecific crosses in subgenus *Passiflora*, the observed numbers and relative frequencies of paternal, maternal and biparental plastid inheritance were 266 (~75%), 43 (~12%) and 48 (~13%), respectively.

Based on six interspecific crosses in subgenus *Decaloba,* maternal and/or biparental inheritance predominated in most hybrid crosses, with rare paternal inheritance (Table 1). Two hybrid crosses, *P. organensis* × *P. biflora* and *P. microstipula* × *P. lancetillensis,* exhibited solely maternal inheritance in all the embryos examined, whereas maternal together with biparental inheritance was detected in three hybrid crosses, *P. rufa* × *P. jatunsachensis, P. rufa* × *P. auriculata* and *P. misera* (9023) × *P. misera* (9335). Within subgenus *Decaloba,* paternal inheritance was only detected in two of 10 progeny of the *P. lancetillensis* × *P. microstipula* cross, while the remaining eight displayed biparental inheritance. The observed numbers and relative frequencies of paternal, maternal and biparental plastid inheritance for the 59 embryos examined in subgenus *Decaloba* were 2 (~3%), 31 (~53%) and 26 (~44%), respectively. An interspecific cross, *P. sphaerocarpa* × *P. pittieri* from subgenus *Astrophea*, had predominantly paternal inheritance, with maternal inheritance detected in only a single embryo. A statistical test was performed to examine whether the patterns of inheritance were related to the subgenus that the parents belong to, *Passiflora* versus *Decaloba*. The estimated χ^2^ value (113.13) for the χ^2^ contingency test was substantially greater than the critical value (χ^2^ = 5.99) at a significance level of 0.05 with 2 degrees of freedom. Therefore, the null hypothesis that the pattern of plastid inheritance is independent of subgenera was rejected (*P* value = 2.2 × 10^−16^). Instead, subgenus dependent patterns of plastid inheritance were detected in subgenera *Passiflora* and *Decaloba.*

**Figure 2:**
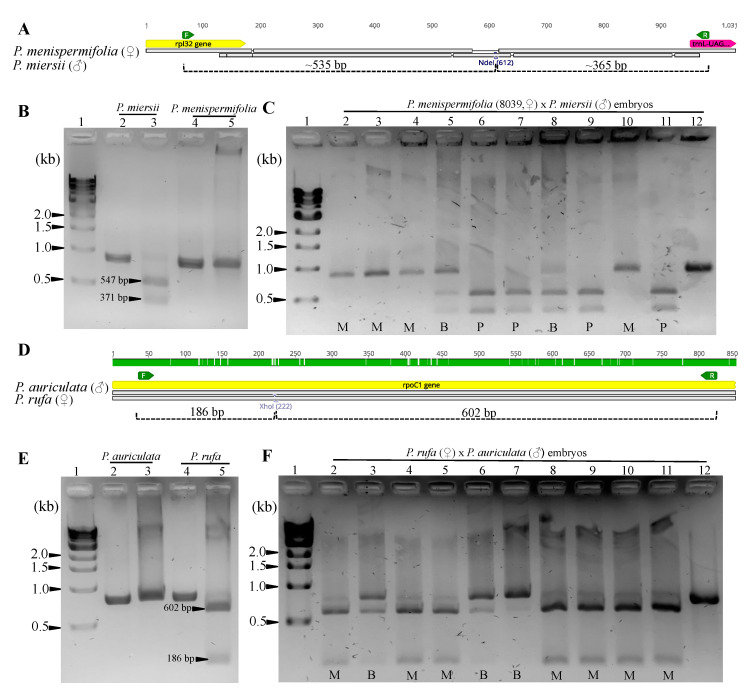
Agarose gels of PCR amplified target regions following digestion with restriction endonucleases from *Passiflora* hybrid embryos. (**A**) The intergenic region (*rpl32-trnL*) amplified from *P. menispermifolia* and *P. miersii* and their hybrid (1044) with estimated fragment sizes after NdeI digestion. (**B**) Lanes 2 and 4 are amplicons of *rpl32-trnL* for *P. miersii* and *P. menispermifolia* and lanes 3 and 5 are products after digestion with NdeI at 37 °C for 12 h. (**C**). Lanes 2–11 are NdeI digestion (at 37 °C for 12 h) products of amplified *rpl32-trnL* for 10 embryos of hybrid 1044. Lane 12 is undigested PCR product for embryo 1 of hybrid 1044 as a reference. (**D**) The partial coding region within *rpoC1* amplified from *P. rufa* and *P. auriculata* and their hybrid progeny (2005) with estimated fragment sizes after XhoI digestion. (**E**) Lanes 2 and 4 are amplicons of *rpoC1* for *P. auriculata* and *P. rufa* and lanes 3 and 5 are products after digestion with XhoI at 37 °C for 12 h. (**F**) Lanes 2–11 are XhoI digestion (at 37 °C for 12 h) products of amplified *rpoC1* for 10 embryos of 2005. Lane 12 is undigested PCR product for embryo 1 of hybrid 2005 as a reference. Lane 1 in all gel images (**B**,**C**,**E**,**F**) contains one kb DNA ladder (NEB). DNA bands were separated in 1.5% agarose gel stained with RedSafe^TM^ (INtRON Biotechnology). Abbreviations: P—Paternal, M—Maternal and B—Biparental.

Agarose gels of RE digested amplicons representing examples of paternal, maternal and biparental inheritance detected in the hybrid embryos are shown in Figure 2, including hybrids of *P. menispermifolia* × *P. miersii* representing subgenus *Passiflora* (Figure 2A–C) and *P. rufa* × *P. auriculata* from subgenus *Decaloba* (Figure 2B,D–F). Gel images of RE digestion for all parent species and hybrid embryos are shown in Supplementary Figures S1 and S2, respectively.

### 2.2. Plastid Inheritance in Seedlings and Maturing Leaves

Plastid inheritance was assessed using tissues from hybrids, including cotyledons, first leaves and subsequent leaves, to detect the plastid type at various stages of plant development. When biparental plastid inheritance was detected in the cotyledon and the first leaf, plastid assessment was continued using subsequent emerging leaves in surviving plants. Where uniparental plastid inheritance was detected in cotyledons and the first leaf, no further assessments were carried out.

Seed germination for most hybrid crosses was substantially lower relative to the number of seeds produced. As a consequence, the total number of interspecific crosses available for further analyses was limited to 10 in subgenus *Passiflora* and seven in subgenus *Decaloba* including a reciprocal cross (Table 2). Within subgenus *Passiflora*, paternal inheritance was detected in the green tissues of all hybrid progeny assessed (Supplementary Figure S3). The crosses that displayed paternal inheritance in the embryos also showed paternal inheritance in the cotyledons of siblings. The hybrid progeny of *P. menispermifolia* (8039) × *P. hastifolia*, *P. nephrodes* × *P. sprucei* and *P. kermesina* × *P. miersii* displayed solely paternal inheritance in the seedlings, although the progeny of these three crosses had maternal and biparental inheritance in addition to paternal inheritance in embryos.

**Figure 3:**
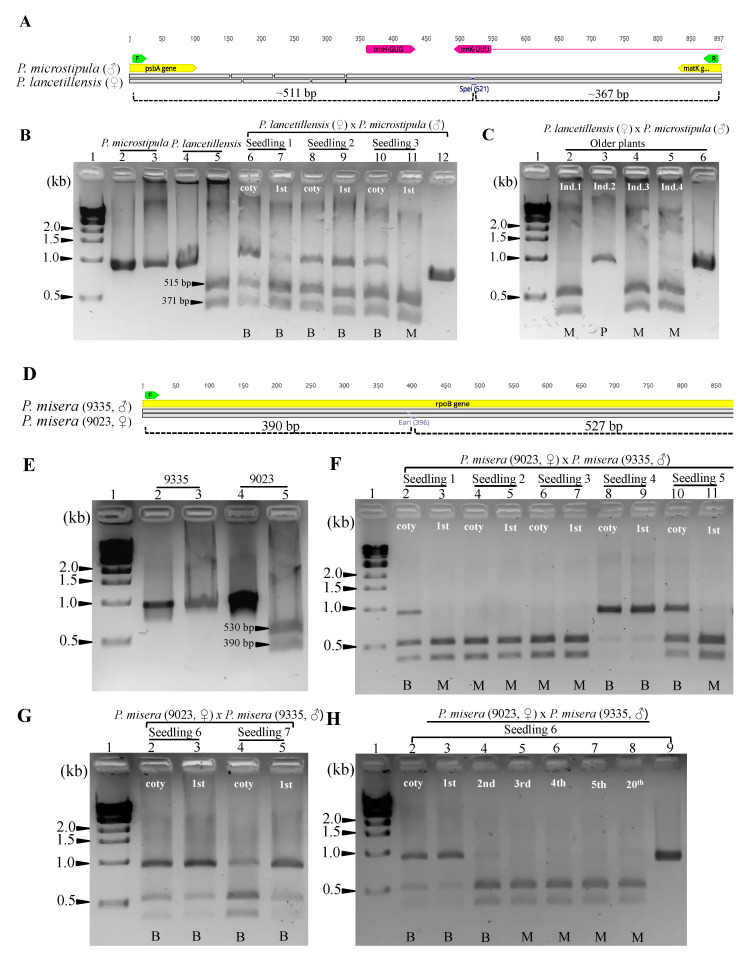
Agarose gels of PCR amplified target regions following digestion with restriction endonucleases from *Passiflora* hybrid seedlings and older leaves. (**A**) The intergenic region (*psbA-matK*) amplified from *P. lancetillensis* and *P. microstipula* and their hybrids (2031) with estimated fragment sizes after SpeI digestion. (**B**) Lanes 2 and 4 are amplicons of *psbA-matK* from *P. microstipula* and *P. lancetillensis* and lanes 3 and 5 are products after SpeI digestion at 37 °C for 12 h. Lanes 6–11 are SpeI digested (37 °C for 12 h) PCR products amplified from hybrid seedlings (2031). For each seedling, the digested PCR products are shown for cotyledon (coty) and the first leaf (1st). Lane 12, undigested PCR product of 2031 seedling 1 (cotyledon) as a reference. (**C**) Lanes 2–5, SpeI digestion products using mature leaves for four hybrid (2031) individuals (Ind.) that were not included in the seedlings analyses. Lane 6, undigested PCR product for 2031 Ind.1. (**D**) The partial coding region within *rpoB* amplified from *P. misera* 9023 and *P. misera* 9355 and their hybrid progeny (2027) with estimated fragment sizes after EarI digestion. (**E**) Lanes 2 and 4 are amplicons of the *rpoB* fragment for *P. misera* 9355 and *P. misera* 9023 and lanes 3 and 5 are products after EarI digestion at 37 °C for 12 h. (**F**) Lanes 2–11, EarI digested (37 °C for 12 h) PCR product for five hybrid seedlings (2027) of *P. misera* 9023 × *P. misera* 9355. For each seedling, the digested products are shown for cotyledon (coty) and the first leaf (1st^‘^). Lane 12, undigested PCR product amplified from 2027 seedling 1 (cotyledon). (**G**) Lanes 2–5, EarI digested PCR products for two additional hybrid seedlings of 2027, in the same order as **F**. (**H**) Lanes 2–8, EarI digested PCR products for seedling 6 of 2027 at different developmental stages, coty—cotyledons, 1st—first leaf, 2nd—second leaf, 3rd—third leaf, 4th—fourth leaf, 5th—fifth leaf, 20th—20th leaf. Lane 9, undigested PCR product of 2027 seedling 6 (20th leaf) as a reference. Lane 1 in all gel images (**B**,**C**,**E**,**H**) contains one kb DNA ladder (NEB). DNA bands were separated in 2% agarose gel and visualized with RedSafe^TM^ (INtRON Biotechnology). Abbreviations: P—Paternal, M—Maternal and B—Biparental.

Hybrid progeny from subgenus *Decaloba* crosses showed all three patterns of plastid inheritance. Most hybrids displayed predominantly maternal inheritance and biparental inheritance, with rarely paternal inheritance. Exclusive maternal inheritance was detected in all seedlings for two crosses, *P. organensis* × *P. biflora* and *P. microstipula* × *P. lancetillensis*, which was consistent with solely maternal inheritance detected in embryos (Figure S3). The hybrids *P. rufa* × *P. auriculata* and *P. misera* (9023) × *P. misera* (9335) displayed uniparental and biparental inheritance in the seedlings, again consistent with embryonic inheritance patterns. Biparental inheritance was detected in the cotyledons for all three hybrid seedlings analyzed for the cross *P. lancetillensis* × *P. microstipula* (Table 2, Figure 3). Among them, two seedlings retained plastids from both parents in the first leaf, while exclusively maternal plastids were detected in the first leaf of the third seedling (Figure 3B). The two seedlings that retained plastids from both parents died precluding plastid assessment in subsequent leaves. Therefore, plastid inheritance was assessed using the leaves of four one-year-old *P. lancetillensis* × *P. microstipula* hybrids that were generated from an independent cross. In these mature *P. lancetillensis* × *P. microstipula* hybrids, maternal plastids were detected in three individuals and paternal in one individual (Figure 3C).

An assessment of plastid inheritance across developmental stages was carried out for seven hybrid progeny of the cross *P. misera* (9023) × *P. misera* (9335) (designated seedlings 1 through 7; Figure 3E–H). Among the seedlings examined, five inherited plastids biparentally (seedlings 1, 4, 5, 6 and 7) and two inherited maternal plastids in their cotyledons (seedlings 2–3). When the first leaves were examined, two individuals (seedlings 1 and 5) appeared to have excluded paternal plastids, while plastids from both parents were detected in the remaining three seedlings (Table 2, Figure 3F,G). Among the three seedlings showing biparental inheritance (seedlings 4, 6 and 7), seedling 4 displayed strongly amplified bands for paternal plastids compared to the remaining two seedlings (Figure 3F). Subsequent examination of plastid types in the second and third leaves of seedling 4 showed the retention of only the paternal plastid type. In contrast, plastid types from both parents were detected up to the second leaf in seedling 6, but only the maternal plastid type was detected in leaf 3 and in leaves of mature plants (Figure 3H). Similar observations, the presence of plastids from both parents up to the second leaf and the retention of maternal plastids afterwards were also noted for seedling 7.

**Figure 4:**
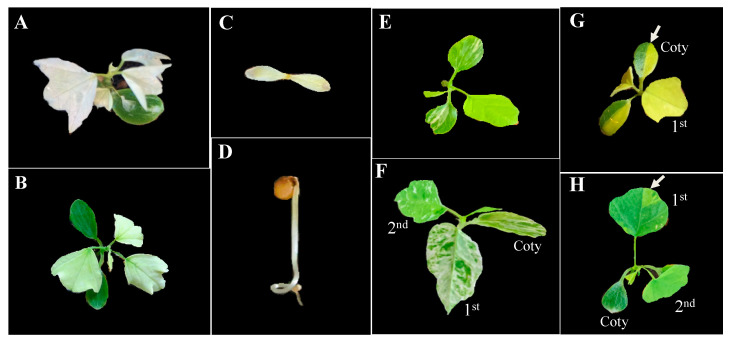
*Passiflora* hybrid seedling phenotypes. (**A**,**B**) *P. organensis* x *P. biflora* hybrids. The hybrids displayed green cotyledons with white (**A**) or pale green (**B**) leaves; all hybrids died within a few weeks. (**C**,**D**) *P. rufa* × *P. auriculata* hybrids. The hybrids produce white/pale cotyledons (**C**) or hypocotyl (**D**) and die before developing leaves. (**E**) *P. microstipula* × *P. lancetillensis* hybrid. Variegation was observed in cotyledons. (**F**) *P. lancetillensis* × *P. microstipula*. Variegation was observed in cotyledons as well as few early leaves in the seedling. (**G**,**H**) *P. misera* (9023) × *P. misera* (9335) hybrids. Variegation was noted in cotyledons and occasionally in first leaf in some seedlings. The variegation phenotype often coordinates with vegetative segregation of biparentally inherited plastids detected through molecular analysis. Agarose gel images for seedlings 1 and 6 in Figure 3F,H are associated with seedling images G and H in this figure, respectively.

Hybrids that displayed incompatibility phenotypes were primarily restricted to subgenus *Decaloba*, with a single case observed in subgenus *Passiflora.* Hybrid progeny of *P. menispermifolia* (8039) × *P. hastifolia* from subgenus *Passiflora* exhibited a dwarf phenotype with severely reduced leaves and altered leaf morphology (Supplementary Figure S4. Within subgenus *Decaloba*, all progeny of *P. organensis* × *P. biflora* had normal green cotyledons but displayed white or pale green leaves that died within a few weeks (Figure 4A,B). Hybrid lethality was also observed in two hybrid crosses, *P. rufa* × *P. auriculata* and *P. rufa* × *P. jatunsachensis*. Both crosses produced albino seedlings that died without producing cotyledons in some progeny (Figure 4C,D). A variegated phenotype was observed for *P. microstipula* × *P. lancetillensis* only in the cotyledons (Figure 4E), whereas their reciprocal cross, *P. lancetillensis* × *P. microstipula*, displayed variegation in the cotyledons as well as in leaves, where white sectors gradually reduced as the plant developed (Figure 4F). Similarly, hybrid variegation was also observed in *P. misera* (9023) × *P. misera* (9335), mostly in the cotyledons and occasionally in the first leaf (Figure 4G,H).

## 3. Discussion

The widespread misconception that plastomes are strictly maternally inherited has major implications for several fields of study, including phylogenetic inference, population genetics, plastome genetic engineering and the consequences of cytonuclear incompatibilities [17,43,51,52]. The presence of ptDNA in generative or sperm cells indicates the potential for transmission of paternal plastids, and the staining of ptDNA in pollen grains has been used to examine plastid inheritance in a large group of angiosperms [7,8]. However, this method does not confirm that paternal plastids are transmitted into a zygote, as multiple mechanisms facilitate paternal plastid exclusion during gametogenesis, fertilization and post fertilization [3,4,11,12,13,53]. Low frequency transmission of paternal plastids is also susceptible to random drift, reducing heteroplasmy during embryo development [3,30]. Similarly, biparental plastid inheritance detectable as heteroplasmy during early plant development has been shown to sort-out, resulting in homoplasmy at later stages [3,4,25]. This suggests that the examination of plastid types in mature plants will likely detect retained plastids and may not reflect how plastids are initially transmitted.

In this study, plastid inheritance was investigated in *Passiflora* hybrids by assaying embryos, cotyledons and leaves to capture inheritance and retention of plastids at different developmental stages. To assess the plastid types in hybrids, RE digestion of PCR amplicons was performed, a method utilized in previous studies of other angiosperms [34,35,54]. While this approach may over-represent uniparental inheritance, it is unlikely to do so with respect to assignments of biparental inheritance. Trace amounts of ptDNA transmitted by one parent could fail to amplify, resulting in an incorrect characterization of plastid inheritance as uniparental. The incomplete digestion of amplicons could over-estimate biparental inheritance; however, incubation times for restriction digestion were extended for 12 h or longer in most cases. Extended incubation times resulted in complete digestion for most *Passiflora* parent species, with very faint undigested product noted in a few parents (Figure 2B,E, Supplementary Figures S1.2, S1.3 and S1.5). The faint undigested band, as noted in the parents, was taken into consideration while assigning the pattern of inheritance in their hybrid progeny. Therefore, in a few cases, despite the presence of faint undigested bands in the hybrids, the pattern of plastid inheritance was considered uniparental.

### 3.1. Clade-Specific Patterns of Inheritance

Based on available data, each subgenus within *Passiflora* displayed a distinct pattern of plastid inheritance, which can be roughly summarized as: (i) predominant paternal inheritance with occasional maternal and biparental inheritance in subgenus *Passiflora*, (ii) predominant maternal and biparental inheritance with rare solely paternal inheritance in subgenus *Decaloba,* and (iii) predominant paternal inheritance with rare maternal inheritance in subgenus *Astrophea* (Table 1 and Table 2). The single interspecific cross in subgenus *Astrophea* precludes the generalization of paternal plastid inheritance as a predominant pattern within the subgenus. The dichotomy of predominant paternal inheritance as a general pattern in subgenus *Passiflora* (~75% progeny) and maternal inheritance in subgenus *Decaloba* (~53% progeny) is in agreement with the previous observation based on a few interspecific hybrids [48]. Paternal transmissions are commonly observed in interspecific crosses or crosses that include divergent parents within a population or geographically isolated populations and may be attributed to failure in the mechanisms to prevent paternal leakage [14,17,34,35,53,55]. While paternal inheritance in subgenus *Passiflora* supports the correlation between paternal inheritance and interspecific crosses, the predominant maternal and biparental inheritance observed for the interspecific hybrids in subgenus *Decaloba* contradicts this phenomenon. In subgenus *Decaloba,* maternal inheritance was the predominant pattern (~53%) in interspecific hybrid embryos, as it was in a previously characterized intraspecific hybrid [29], suggesting that uniparental-maternal is the dominant pattern of inheritance in *Decaloba* in nature.

The crosses between *P. misera* (9023) and *P. misera* (9335) in this study were considered interspecific as the two *P. misera* are very distinct in morphology of flowers, leaves and stems (Supplementary Figure S4). *Passiflora misera* is known for its conspicuously flattened stems lacking showy flowers [56], but the species is highly variable and it was suggested that multiple species may be represented under the name *P. misera* [57]. Since the two accessions of *P. misera* were treated as distinct species, the present study did not consider intraspecific crosses in *Passiflora*.

Paternal inheritance is an anomaly among angiosperms, but cases have been reported in a few lineages including *Actinidia, Turnera* and *Medicago* [26,27,36,58]. The predominance of paternal plastid inheritance observed in hybrids of subgenus *Passiflora* is analogous to intraspecific crosses in *Turnera* [26,27], which is now classified in the Passifloraceae subfamily Turneroideae [59]. Intraspecific crosses in *Turnera* displayed predominant paternal with occasional maternal or biparental inheritance. It is possible that intraspecific crosses in subgenus *Passiflora* also follow paternally biased plastid inheritance, and thus represent one of the few angiosperm lineages with predominant paternal inheritance. Evaluating plastid inheritance in intraspecific crosses of subgenus *Passiflora* could help illuminate this hypothesis. The similarity in inheritance patterns in subgenus *Passiflora* and *Turnera* suggests that paternal inheritance may be the ancestral condition in Passifloraceae, regardless of whether the cross is within or between species. There may have been a shift towards predominantly maternal inheritance in the genus, specifically in subgenus *Decaloba.* A single interspecific hybrid from subgenus *Astrophea* also shows predominantly paternal inheritance similar to species in subgenus *Passiflora.* However, the result should be cautiously interpreted as a general pattern of inheritance for subgenus *Astrophea* because of the small sample size. The hypothesis of paternal and maternal inheritance associated with inter- and intraspecific crosses, respectively, in *Passiflora* requires evidence from intraspecific crosses. Interspecific crosses suggest that hybrids in subgenus *Passiflora* exhibit predominant paternal plastid inheritance, whereas hybrids in subgenus *Decaloba* predominantly inherit maternal plastids.

### 3.2. Occurrence of Biparental Plastid Inheritance

Two separate studies presented molecular evidence of biparental inheritance in *Passiflora.* One study examined reciprocal interspecific crosses between *P. menispermifolia* and *P. oerstedii* (subgenus *Passiflora*) [50], and the other an intraspecific cross of *P. costaricensis* (subgenus *Decaloba*) from two geographical locations [29]. The hybrids of *P. menispermifolia* and *P. oerstedii* displayed a bleached phenotype with white and green sectors in leaves containing plastomes of *P. menispermifolia* and *P. oerstedii*, respectively, and non-differentiated plastids of *P. menispermifolia* indicated incompatibility with hybrid nuclear genotype [50]. In contrast, Hansen et al. (2007) [50] did not report biparental inheritance but instead found strictly paternal inheritance in *P. menispermifolia* x *P. oerstedii* hybrids as well as in backcross experiments. It is noteworthy that the *P. oerstedii* illustrated in Mráček (2005) [29] has deeply trilobed leaves that differ from commonly described *P. oerstedii* with oblong-ovate simple leaves included in Hansen et al. (2007). Ulmer and MacDougal (2004) suggested that two species, *P. oerstedii* var. *choconiana* (also known as *P. choconiana*) and *P. purpusii* with trilobed leaves are erroneously identified under the name *P. oerstedii* [49]. It is likely that the difference in plastid inheritance noted in these two studies is due to incorrect identification of the parent species.

In the present study, in crosses that employed *P. menispermifolia* and *P. oerstedii* as parents, the results depended on varieties being used. The hybrids between *P. menispermifolia* (9224) and *P. oerstedii* inherited plastids from *P. menispermifolia* (9224) regardless of the cross direction. However, when the cross included a different accession of *P. menispermifolia* (8039), biparental plastid inheritance was detected in all hybrid embryos (Table 1, Supplementary Figure S2). In general, the frequent inheritance of *P. menispermifolia* plastids was detected in crosses that included *P. menispermifolia* either as a paternal or maternal parent (Table 1). Plastome size variation may be worth considering as an explanation for this result. The *P. menispermifolia* plastome is substantially smaller (~13 kilobases) than other species in subgenus *Passiflora* [60] and this difference may play a role in efficient plastome replication, resulting in an advantage over the other species during plastid transmission. It has been proposed that the speed of plastid multiplication plays a role in the transmission of plastids in *Oenothera* [22].

Heteroplasmy due to biparental inheritance was detected in the embryos and seedlings of *Passiflora* hybrids but not in mature plants, which is a clear distinction from previous findings. During plant development, biparentally inherited plastids in *Decaloba* hybrids segregated to exclude either parental plastid type, resulting in homoplasmy. This observation is very similar to the segregation of biparentally inherited plastids in *Medicago* [25]. Vegetative segregation in *Decaloba* hybrids was complete in the F1 generation, similar to most *Medicago* hybrids. The sorting-out of plastid types observed in *Passiflora* and *Medicago* supports the prediction that the completion of vegetative segregation occurs within a generation [4]. In fact, homoplasmy in *Decaloba* hybrids was usually attained in the first leaf, but in some seedlings, the process was not complete until the second or third leaf, indicating that the rate of segregation differs among hybrid individuals (Figure 3F,H). Of seven progeny examined for *P. misera* (9023) × *P. misera* (9335), only one retained paternal ptDNA in the leaves of mature plants (Figure 3F,G), while the remaining resolved to maternal plastid types.

Vegetative segregation strongly favoring the maternal plastid type was reported in *Medicago* [25]. Mechanisms controlling plastome replication and partitioning within the plastid prior to organelle fission, and ultimately plastid partitioning at cell division, are not well understood. The relative abundance of parental plastids and their plastomes present early in the formation of hybrid progeny may favor vegetative segregation toward homoplasmy for one type or the other, in the absence of selection [2,3,4]. This suggests that the resolution to homoplasmy for the maternal type may be stochastic in *Decaloba* hybrids. It is possible that selection plays a role in the preference for the maternal plastid. However, the limited number of crosses and progeny considered here did not allow a conclusion regarding whether the preference for the maternal plastid type is due to selection or random processes. Nonetheless, the vegetative segregation of biparentally inherited plastids as observed in *Decaloba* seedlings mandates that the patterns of plastid inheritance be examined early in plant development, as the assignment using mature plants may not accurately reflect inheritance patterns.

### 3.3. Hybrid Incompatibility Phenotypes

Hybrid variegation in *Passiflora* seedlings was observed for three crosses, *P. microstipula* × *P. lancetillensis*, *P. lancetillensis* × *P. microstipula* and *P. misera* (9023) × *P. misera* (9335) (Figure 4E–H). Variegation in the cotyledons and leaves of *P. misera* (9023) × *P. misera* (9335) was often coincident with the segregation of biparentally inherited plastids (Figure 3F–H and Figure 4G,H, Table 2). Variegation was observed in the cotyledons of *P. microstipula* x *P. lancetillensis*, but heteroplasmy was not detected in the leaves (Figure 4E, Table 2). Similarly, the reciprocal cross *P. lancetillensis* × *P. microstipula* displayed variegation in cotyledons as well as leaves, and the white sectors gradually reduced as the plants developed (Figure 4F, Supplementary Figure S4). Heteroplasmy detected in *P. lancetillensis* × *P. microstipula* hybrids was mostly confined in cotyledons and not observed in the mature leaves (Figure 3B,C, Table 2). This suggests that the variegation observed in the leaves of *P. lancetillensis* and *P. microstipula* hybrids may not be associated with the segregation of plastids, but could be mediated by another mechanism under nuclear influence [1,61]. Many species from subgenus *Decaloba* are known for conspicuous leaf variegation in younger and/or mature plants [47,49]. Additional evidence is needed to confirm if hybrid variegation is associated with vegetative segregation in the genus.

*Passiflora* hybrids that displayed incompatibility phenotypes were mostly within subgenus *Decaloba.* Hybrid incompatibility resulted in inviable hybrids for three crosses, *P. organensis* × *P. biflora*, *P. rufa* × *P. jatunsachensis* and *P. rufa* × *P. auriculata* (Figure 4A–D, Supplementary Figure S4). Progeny of *P. rufa* × *P. auriculata* inherited uniparental (maternal or paternal) or biparental plastids, and regardless of the pattern of inheritance, all progeny displayed an albino phenotype and failed to produce leaves (Figure 4C,D, Supplementary Figure S4). The white/pale phenotypes in hybrids are generally associated with lower chlorophyll content, delayed thylakoid development and reduced photosynthesis [62]. In these hybrids, the inviability observed was most likely under the influence of the nuclear genome as the phenotype was consistent regardless of the plastid inheritance pattern. A similar phenotype with albino/pale green leaves was observed in *P. organensis* × *P. biflora* hybrids that inherited only maternal plastids in all progeny, although the hybrids produced green cotyledons. This suggests that the phenotype may have been associated with leaf development due to the improper assembly of photosynthetic components, possibly due to incompatible interactions between plastid and nuclear genomes [62]. It has been suggested that postzygotic reproductive barriers can be generally predicted from the observed phenotype [63]. Postzygotic barriers that lead to hybrid inviability usually arise when co-adapted loci accumulate mutations independently in their lineages and the subsequent reunion of the incompatible alleles in hybrids results in a negative interaction, as explained by the Dobzhansky-Muller model (reviewed in [63,64]). This model has been further extended for co-adapted loci in two cellular compartments, nucleus and plastid, which are disrupted in hybrids, leading to plastome-genome incompatibility [62]. The availability of complete plastomes for *Passiflora* hybrids that displayed the incompatibility phenotype could be used to identify underlying plastome regulatory elements associated with hybrid incompatibility.

## 4. Conclusions

Clade-specific plastid inheritance along with the occurrence of biparental inheritance makes *Passiflora* an intriguing system to study the underlying evolutionary mechanisms associated with plastid inheritance. The diverse inheritance patterns within a single genus suggest that multiple evolutionary forces are in play. Biparental inheritance is thought to be advantageous for rescuing defective plastids and slowing down the accumulation of mutations through strictly uniparental inheritance in angiosperms [15,16,17]. This may explain the instances of biparental inheritance and paternal/maternal leakage detected in *Passiflora*, but is not adequate to explain predominant paternal inheritance in subgenus *Passiflora.* The detection of paternally biased plastid transmission, also observed in the closely related subfamily Turneroideae, suggests that paternal inheritance may be the ancestral condition in these lineages.

Self-incompatibility is common in *Passiflora* and species can readily outcross, yielding viable hybrids that can be backcrossed to either parent [29,49] and also with other species generating viable new hybrids in greenhouse experiments (B.S. and L.E.G., personal observation). This shows the potential of *Passiflora* species to outcross and likely produce hybrids in the case of natural cross-pollination. The ability to hybridize, coupled with diverse plastid inheritance patterns, could be problematic for resolving phylogenetic relationships. In fact, a conflicting relationship was identified in *Passiflora* phylogenetic studies using heteroplasmic plastome loci, suggesting that researchers should be cautious in interpreting phylogenetic relationships in the genus with plastid data [41]. In the present study, although heteroplasmy was restricted to seedlings and vegetative segregation led to the retention of either maternal or paternal plastid types in later stages of development, this phenomenon could still contribute to conflicting phylogenetic relationships in *Passiflora* [29]. It is notable that the nuclear transfer of plastid genes in *Passiflora* is widespread, and duplicated plastid loci co-existing in two cellular compartments undergo different evolutionary constraints [65]. As a consequence, this may also introduce conflicts associated with paralogy when inferring phylogenetic relationships, another reason to be cautious using plastid loci for phylogeny reconstruction in *Passiflora.*

Multiple patterns of plastid inheritance in *Passiflora* could also play a role in plastome evolution in the genus. *Passiflora* plastomes are highly rearranged with extensive structural variation and highly accelerated substitution rates in certain genes and clades [60,66]. Although recombination between plastomes from different plastids is rare, empirical evidence of plastome recombination has been documented in protoplast fusion experiments [31,67]. Evidence of plastome structural rearrangements correlated with biparental inheritance has been recognized in angiosperms [51] and isogamous algae [68]. It will be interesting to examine whether plastid inheritance patterns have played a role in the unusual rearrangements in *Passiflora* plastomes.

## 5. Materials and Methods

### 5.1. Passiflora Hybrids and Seed Germination

*Passiflora* hybrids were generated from field-collected populations grown in greenhouses at The University of Texas at Austin. Interspecific hybrids were generated from self-incompatible *Passiflora* clones; self-compatible species were excluded from the study. Crosses were made by brushing pollen from flowers of paternal parents onto the stigma of flowers of maternal parents. The anthers of the maternal parents were removed prior to pollen maturation. Crosses were carried out between plants located in separate greenhouses to avoid uncontrolled pollination. Reciprocal crosses were attempted, dependent on the availability of flowers. Two approaches were used for seed germination. Seed coats were scarified and then seeds were soaked in water (25 °C) for 24 h and placed between moist paper towels inside a plastic bag in the dark until seed germination was observed. The germinated seedlings were transferred into Pro-Mix^®^ (Premier Tech, Riviére-du-Loup, QC, Canada) growing medium and moved to the greenhouse. Alternatively, seeds were soaked in water (25 °C) without scarification for 24 h and directly transferred into Pro-Mix^®^ growing medium in the greenhouse.

### 5.2. Amplification of Target Regions and RE Digestion to Assess Plastid Type

Complete plastomes available at The National Center for Biotechnology (NCBI) for 31 *Passiflora* species [60,69] were used to identify regions that contained suitable nucleotide variability. The variable regions were amplified and Sanger sequencing was carried out to determine the sequence for species that lack complete plastome sequences. Primer design for the PCR amplification and Sanger sequencing was carried out with Primer3 [70] in Geneious v. 11.0.5 (https://www.geneious.com (accessed on 20 November 2020)). The newly obtained sequences were aligned with available *Passiflora* plastome data using multiple alignment using fast Fourier transform (MAFFT) [71] in Geneious v.11.0.5. The ptDNA target regions were selected based on the presence of polymorphic restriction sites between the parents.

The target regions for the parents were amplified followed by restriction enzyme (RE) digestion, and were visualized in agarose gels. This process was repeated for hybrids. Banding patterns of digested amplicons from parents post digestion were used as references to assess plastid types in hybrids. Restriction digests of amplified ptDNA were performed for variable time periods at the optimal temperature recommended by the RE suppliers (New England Biolabs, Ipswich, MA, USA and Thermo Scientific, Waltham, MA, USA). The incubation time for digestion was held constant for parent species and their hybrids. Digests of amplicons were conducted without prior purification of the PCR products and visualized in 1–2% agarose gels stained with RedSafe^TM^ (iNtRON Biotechnology, Seongnam). One kb DNA ladder (N3232L, New England Biolabs) was used as a marker to estimate amplicon and fragment sizes for the PCR products prior to and post digestion.

### 5.3. Direct PCR Amplification Using Crude Embryo Extract

*Passiflora* hybrid seeds were soaked in water for 24 h and embryos were excised by removing the seed coat and endosperm under a dissecting microscope. The excised embryos were rinsed in sterile water and stored dry at −80 °C until further use. Stored embryos were suspended in 88 μL of 50 mM NaOH and homogenized for 1 min in the presence of five to six 1 mm glass beads (BioSpec Inc., Bartlesville, OK, USA) using Mini-BeadBeater-96 (Glen Mills Inc., Clifton, NJ, USA). The disrupted embryos were incubated at 95 °C for 10 min and neutralized by adding 12 μL of 1 M Tris-HCl (pH 8.0). The crude extract was vortexed, briefly centrifuged and 5 μL of supernatant was used for PCR.

Plastid inheritance in hybrid embryos was assessed by sampling 10 seeds per cross for 44 interspecific crosses. The PCR amplification of crude embryo extract was carried out using Seed-Direct^TM^ PCR mix (D300, Lamda Biotech, St. Louis, MO, USA). The PCR reaction contained 5 μL of crude extract, 10 μL of 2× Seed-Direct^TM^ PCR mix, 1 μL of 10 μM forward and reverse primers and 7 μL of H_2_O. The PCR program included 5 min at 95 °C, followed by 34 cycles of 30 s at 95 °C, 30 s at 52–56 °C, and 1 min at 72 °C with final extension of 5 min at 72 °C. Annealing temperatures were adjusted according to the melting temperature of the different primer sets.

### 5.4. DNA Isolation and PCR Amplification

Total genomic DNA was isolated from young leaves of parents, cotyledons and leaves of hybrid seedlings, and also from hybrid embryos that failed in the direct PCR amplification. Hybrid cotyledons were collected at first leaf emergence, and subsequently, the first leaf was collected after second leaf emergence. This process continued as long as hybrids survived and produced new leaves. For some hybrids, tissues were not collected at the seedling stage and plastid inheritance was assessed exclusively using leaves from one-year old plants, hereafter referred to as mature plants. The tissues were flash frozen in liquid nitrogen and homogenized using Geno Grinder ^TM^ (SPEX, Metuchen, NJ, USA) in the presence of 2–3 metallic beads at 1200 rpm for 15 s. Total genomic DNA was isolated using NucleoSpin^®^ plant II DNA extraction kit (MACHEREY-NAGEL, Düren, Germany). Except for the embryos, qualitative and quantitative assessments of isolated DNA were carried out using agarose gel electrophoresis and NanoDrop^®^ (ND-1000, Thermo Scientific, Waltham, MA, USA). 

Isolated DNA was used for PCR amplification in a final reaction volume of 25 μL that contained approximately 200 ng of DNA, 10 μL of FailSafe^TM^ PCR PreMix D (FSP995D, Lucigen, Middleton, WI, USA), 1 μL of 10 μM forward and reverse primers, 0.5 μL of Taq polymerase and 5–10 μL of H_2_O. The PCR program included 5 min at 95 °C, followed by 34 cycles of 30 s at 95 °C, 30 s at 52–56 °C, and 1 min at 72 °C with final extension of 5 min at 72 °C. The annealing temperature of the PCR cycle was adjusted according to the melting temperature of the different primer sets.

### 5.5. Statistical Analyses

To test the statistical difference in the patterns of plastid inheritance between the groups (subgenera *Passiflora* and *Decaloba*), Chi-square (χ^2^) test statistic was calculated using embryo plastid inheritance data in R v3.5.1 (R Core Team, 2013). The χ^2^ contingency test was carried out to test the null hypothesis that the patterns of inheritance (paternal, maternal and biparental) are independent of the subgenera.

## Figures and Tables

**Table 1:** Plastid inheritance in embryos of *Passiflora* hybrids. Grey highlight indicates reciprocal crosses.

Subgenus	Cross (♀ × ♂)	Accession	Inheritance		
			Paternal	Maternal	Biparental
*Passiflora*	*P. retipetala × P. oerstedii*	1015	10	-	-
	*P. racemosa × P. oerstedii*	1074	10	-	-
	*P. quadrangularis × P. oerstedii*	1075	10	-	-
	*P. sprucei × P. oerstedii*	1014	8	-	-
	*P. oerstedii × P. sprucei*	1063	10	-	-
	*P. nephrodes × P. sprucei*	1061	9	1	-
	*P. choconiana × P. sprucei*	1062	-	5	5
	*P. speciosa × P. sprucei*	1060	10	-	-
	*P. hastifolia × P. miersii*	1003	10	-	-
	*P. oerstedii × P. miersii*	1041	10	-	-
	*P. menispermifolia* (8039) *× P. miersii*	1044	4	4	2
	*P. garckei × P. choconiana*	1019	10	-	-
	*P. quadrangularis × P. garckei*	1054	1	-	-
	*P. oerstedii × P. nephrodes*	1002	10	-	-
	*P. menispermifolia* (9224) *× P. oerstedii*	1004		10	-
	*P. oerstedii × P. menispermifolia* (9244)	1006	10	-	-
	*P. oerstedii × P. menispermifolia* (8039)	1031	-	-	10
	*P. vitifolia × P. hastifolia*	1032	10	-	-
	*P. vitifolia × P. quadrangularis*	1078	10	-	-
	*P. vitifolia × P. edulis*	1077	10	-	-
	*P. vitifolia × P. speciosa*	1053	10	-	-
	*P. menispermifolia* (8039) *× P. hastifolia*	1028	4	-	6
	*P. hastifolia × P. menispermifolia* (8039)	1030	-	-	10
	*P. retipetala × P. amethystina*	1072	10	-	-
	*P. retipetala × P. menispermifolia* (Sirena)	1036	9	-	-
	*P. nephrodes × P. choconiana*	1012	-	9	-
	*P. choconiana × P. nephrodes*	1013	10	-	-
	*P. menispermifolia* (8039) *× P. nephrodes*	1059	-	3	7
	*P. choconiana × P. menispermifolia* (9244)	1008	9	-	1
	*P. choconiana × P. menispermifolia* (Sirena)	1033	10	-	-
	*P. kermesina × P. miersii*	1040	9	1	-
	*P. kermesina × P. hastifolia*	1023	10	-	-
	*P. sprucei × P. retipetala*	1046	10	-	-
	*P. menispermifolia* (8039) *× P. resticulata*	1068	-	3	7
	*P. nitida × P. quadrangularis*	1080	3	7	-
	*P. speciosa × P. polsea*	1073	10	-	-
	*P. miersii × P. amethystina*	1071	10	-	-
*Decaloba*	*P. rufra × P. jatunsachensis*	2001	-	1	9
	*P. rufra × P. auriculata*	2005	-	7	3
	*P. organensis × P. biflora*	2014	-	10	-
	*P. misera* (9023) *× P. misera* (9335)	2027	-	3	6
	*P. microstipula × P. lancetillensis*	2028	-	10	-
	*P. lancetillensis × P. microstipula*	2031	2	-	8
*Astrophea*	*P. sphaerocarpa × P. pittieri*	3002	8	1	-

**Table 2:** Plastid inheritance in seedlings and/or mature plants of *Passiflora* hybrids. Asterisk denotes seedlings that occasionally produced cotyledons and died. Sampling from mature plant is highlighted in grey. Dash indicates the tissues were not sampled for plastid assessment. Abbreviations: P—Paternal, M—Maternal, B—Biparental and NA—tissues not available.

Subgenus	Cross (♀ × ♂)	Accession	Tissues			
			Embryos	Seedlings		Mature Plant Leaf
				Cotyledons	1st Leaf	
*Passiflora*	*P. vitifolia × P. speciosa*	1053	10P	5P	5P	-
	*P. vitifolia × P. edulis*	1077	10P	5P	5P	-
	*P. retipetala × P. amethystina*	1072	10P	4P	4P	-
	*P. miersii × P. amethystina*	1071	10P	2P	2P	-
	*P. vitifolia × P. quadrangularis*	1078	10P	2P	2P	-
	*P. menispermifolia* (8039) *× P. hastifolia*	1028	4P/6B	3P	-	3P
	*P. speciosa × P. sprucei*	1060	10P	3P	3P	-
	*P. nephrodes × P. sprucei*	1061	9P/1M	1P	1P	-
	*P. oerstedii × P. sprucei*	1063	10P	NA	NA	2P
	*P. kermesina × P. miersii*	1040	9P/1M	NA	NA	2P
*Decaloba*	*P. organensis × P. biflora*	2014	10M	6M	6M	-
	P. rufra × P. auriculata *	2005	7M/3B	1M/1P/1B	NA	NA
	*P. microstipula × P. lancetillensis*	2028	10M	5M	5M	-
	*P. lancetillensis × P. microstipula*	2031	2P/8B	3B	2B/1M	1M
	*P. lancetillensis × P. microstipula*	2031	NA	NA	NA	3M/1P
	*P. misera* (9335) *× P. misera* (9023)	2018	NA	NA	NA	3M
	*P. misera* (9023) *× P. misera* (9335)	2027	3M/6B	2M/5B	4M/3B	6M/1P

## Data Availability

Data available in a publicly accessible repository. GenBank accession numbers for the new sequences are found in Supplementary Table S4.

## Supplementary Materials

The following are available online at www.mdpi.com/xxx/s1. Table S1. List of *Passiflora* species included in the study with original collection location, voucher ID and GenBank accession number. Table S2. List of *Passiflora* crosses included in the study with the information regarding the ptDNA target regions, restriction enzymes, amplicon size prior to and post digestion, and voucher ID. Table S3. Oligonucleotide primers used for PCR amplification and Sanger sequencing of the target regions. Table S4. GenBank accession numbers (GBN) for the target region sequences generated with Sanger sequencing for *Passiflora* species and their hybrids. Figure S1. Agarose gels of PCR amplified ptDNA regions following restriction digestion from *Passiflora* parents. Figure S2. Agarose gels of PCR amplified ptDNA regions following restriction digestion from *Passiflora* hybrid embryos. Figure S3. Agarose gels of PCR amplified ptDNA regions following restriction digestion from *Passiflora* hybrid seedlings. Figure S4. *Passiflora* hybrid seedlings and their parents.
